# Optimisation and Validation of a conventional ELISA and cut-offs for detecting and quantifying anti-SARS-CoV-2 Spike, RBD, and Nucleoprotein IgG, IgM, and IgA antibodies in Uganda

**DOI:** 10.3389/fimmu.2023.1113194

**Published:** 2023-03-14

**Authors:** Gerald Kevin Oluka, Patricia Namubiru, Laban Kato, Violet Ankunda, Ben Gombe, Matthew Cotten, The COVID-19 Immunoprofiling Team, Claire Baine, Monica Musenero, Pontiano Kaleebu, Julie Fox, Jennifer Serwanga

**Affiliations:** ^1^ Pathogen Genomics, Phenotype, and Immunity Program, Medical Research Council, Uganda Virus Research Institute and London School of Hygiene and Tropical Medicine, Uganda Research Unit, Entebbe, Uganda; ^2^ Department of Immunology, Uganda Virus Research Institute, Entebbe, Uganda; ^3^ Medical Research Council, University of Glasgow Centre for Virus Research, Glasgow, United Kingdom; ^4^ Science, Technology, and Innovation Secretariat, Office of the President, Government of Uganda, Kampala, Uganda; ^5^ Guy’s and St Thomas’ National Health Services Foundation Trust, King’s College London, London, United Kingdom

**Keywords:** SARS-CoV-2, spike-protein, Nucleoprotein, RBD, ELISA validation and optimization, antibody seropositivity cut-offs, Uganda, Sub-Saharan Africa

## Abstract

There is an urgent need for better immunoassays to measure antibody responses as part of immune-surveillance activities and to profile immunological responses to emerging SARS-CoV-2 variants. We optimised and validated an in-house conventional ELISA to identify and quantify SARS-CoV-2 spike- (S-), receptor binding domain- (RBD-), and nucleoprotein- (N-) directed IgG, IgM, and IgA binding antibodies in the Ugandan population and similar settings. Pre- and post-pandemic specimens were used to compare the utility of mean ± 2SD, mean ± 3SD, 4-fold above blanks, bootstrapping, and receiver operating characteristic (ROC) analyses in determining optimal cut-off optical densities at 450 nm (OD) for discriminating between antibody positives and negatives. “Limits of detection” (LOD) and “limits of quantitation” (LOQ) were validated alongside the assay’s uniformity, accuracy, inter-assay and inter-operator precision, and parallelism. With spike-directed sensitivity and specificity of 95.33 and 94.15%, respectively, and nucleoprotein sensitivity and specificity of 82.69 and 79.71%, ROC was chosen as the best method for determining cutoffs. Accuracy measurements were within the expected CV range of 25%. Serum and plasma OD values were highly correlated (r = 0.93, p=0.0001). ROC-derived cut-offs for S-, RBD-, and N-directed IgG, IgM, and IgA were 0.432, 0.356, 0.201 (S), 0.214, 0.350, 0.303 (RBD), and 0.395, 0.229, 0.188 (N). The sensitivity and specificity of the S-IgG cut-off were equivalent to the WHO 20/B770-02 S-IgG reference standard at 100% level. Spike negative IgG, IgM, and IgA ODs corresponded to median antibody concentrations of 1.49, 3.16, and 0 BAU/mL, respectively, consistent with WHO low titre estimates. Anti-spike IgG, IgM, and IgA cut-offs were equivalent to 18.94, 20.06, and 55.08 BAU/mL. For the first time, we provide validated parameters and cut-off criteria for the in-house detection of subclinical SARS-CoV-2 infection and vaccine-elicited binding antibodies in the context of Sub-Saharan Africa and populations with comparable risk factors.

## Introduction

Following the global spread of SARS-CoV-2 and the subsequent emergence of more pathogenic variants, the need for population serosurveillance became urgent to track the course of virus-specific immune responses as a tool for estimating population susceptibility to emerging variants, and informing public health policy about vaccination, immune-surveillance and intervention strategies (1, 2). A well-optimised and validated ELISA is an accurate and cost-effective way to monitor community prevalence and incidence. The results can inform infection control and prevention efforts (3) through comprehensive longitudinal epidemiologic and immuno-surveillance assessments, identification of prior exposure, and assessing the durability of antibodies elicited by COVID-19 vaccines in clinical trials (2). A reliable, sensitive, and specific ELISA also enables accurate detection of convalescent plasma and B-cell donors with binding-antibody titres predictive of virus neutralisation potency (4–6).

While several commercial kits and in-house assays developed elsewhere have become available (7–9), a validated antibody immunoassay that considers different levels of prior cross-reactivity in different populations is needed (10). Several existing commercial assays have shown high levels of inter-assay discordance (11). Comparisons of in-house developed assays and commercial kits have, in some instances, shown in-house assays to have higher specificity and sensitivity (12). Therefore, it is necessary to develop an optimised, well-validated ELISA, which in a Sub-Saharan African setting, would have to factor in the presence of background cross-reactive binding antibodies to other antigens (13, 14). This study underscores the need for population-specific antibody threshold cut-off values to reliably distinguish positive and negative specimens (12, 15).

In this study, we aimed to develop, optimise, and validate an in-house indirect ELISA for detecting and quantifying antibodies directed against the SARS-CoV-2 spike, RBD and nucleoprotein for the Ugandan population and similar settings in Sub-Saharan Africa. The spike (S) and nucleoprotein (N) proteins were of particular emphasis, being the most immunogenic of the SARS-CoV-2 structural proteins (16, 17). Since COVID-19 vaccines contain and elicit antibody responses to the spike protein, antibody responses against the spike protein are often used to evaluate vaccine immunogenicity (17, 18). In addition, antibodies targeting the nucleoprotein imply infection and re-infection (19) in the absence of RT-PCR, as is often the case in low-resource settings in Sub-Saharan Africa, where there is inadequate coverage of molecular diagnostics (20).

## Materials and methods

### Study design and population

This study validated assays for assessing virus-induced antibody responses using serum and plasma specimens from PCR-confirmed SARS-CoV-2 infected subjects initially admitted to COVID-19 isolation hospitals in Masaka or Entebbe. The Uganda Virus Research Institute (UVRI) Research and Ethics Committee (Ref: GC/127/833) and the Uganda National Council of Science and Technology both approved the study as ethical (Ref: HS637ES). To participate in the study, all subjects provided written informed consent.

When this cohort was assembled, all individuals who tested positive for COVID-19 by PCR were immediately admitted to an isolation hospital, regardless of symptom status or disease severity. The date of the first positive PCR or the date of admission was thus used to estimate the approximate timing of the infection. Following admission, participants were followed up on a weekly basis for one month (the acute phase) and then monthly for 24 months (the convalescent phase) to determine the development and durability of virus-specific IgG, IgM, and IgA binding antibodies. Spike- and nucleoprotein protein-directed IgG peaked between 25 and 37 days after infection, IgM peaked between 8 and 12 days, and S-IgA and N-IgA peaked between 7 and 10 days. Positive controls for cut-off value determinations were these primary peak specimens collected during the A23.1 variant wave.

### In-house ELISA for detection of anti-SARS-CoV-2 binding antibodies

We used an indirect in-house ELISA adapted and modified from Pickering et al. (21) to quantify the spike-, RBD- and N-directed IgG, IgM, and IgA binding antibody concentrations (ng/ml) and optical densities at 450 nanometres (OD). Briefly, 96-well flat-bottomed medium-binding plates (Greiner Bio-One, #655001) were coated with 50 μl of N-, S- antigens (R&D Systems #10474-CV-01M, #10549-CV-01M) or wildtype Wuhan RBD-protein (expression plasmid kindly donated by Katie Doores) at three µg/ml (0.15µg per well) in PBS and incubated overnight at 4°C. The plates were then washed 5x with 0.01M PBS containing 0.05% Tween 20 (PBS-T) with a BioTek 405 TS microplate washer and blocked with PBS-T containing 1% BSA (Sigma, #A3803) for 1 hour at room temperature (RT). Heat-inactivated (56˚C for 30 mins) plasma/serum specimens diluted at 1:100 in PBS-T with 1% BSA were added in duplicate and incubated for 2 hours at RT. Following five washes with PBS-T, the plates were incubated with horseradish peroxidase-conjugated, goat anti-human IgG (γ-chain specific, Sigma, #A0170, 1:10,000 dilution), IgM (μ-chain specific, Sigma, #A6907, 1:1,000 for S and 1:5000 for N), or IgA (α-chain specific, Sigma, #A0295, 1:1,000 dilution) detection antibodies in PBS-T containing 1% BSA for 1 hour at room temperature (RT). Pre-determined negative and positive plasma specimens, monoclonal antibodies, CR3009 (2µg/ml) for N or CR3022 (0.1µg/ml) for S, and two sets of duplicate blank wells were included as controls. Finally, the wells were washed and dried by tapping on absorbent paper towels. 50 μl of 3,3′,5,5′-Tetra-methyl benzidine (TMB) substrate (Sera Care, #5120-0075) was then added for 3 minutes, followed by 50 μl of 1M Hydrochloric acid (Sera Care #5150-0021) to stop the reaction. The plates were read at 450nm with a BioTek ELx808 microplate reader using the BioTek GEN5 software. Blank well OD values were subtracted from those in specimen wells to obtain the net response.

### Specimens for determining spike, RBD and nucleoprotein cut-offs

Positive controls were selected from primary peak specimens whose optical densities were higher than the assay’s limit of detection (LOD). We used 107, 77, 54 spike and 112, 51, 46 nucleocapsid specimens, corresponding to the IgG, IgM, and IgA antibody primary peaks, respectively, and summarised in **Table 1**. In addition, retrospective pre-COVID specimens collected between October 2012 and November 2017 for future optimization of immunological assays were used as suitable negative controls, as summarised in **Table 2**.

**Table 1:** Shows the summary statistics of rt-PCR+ subject specimens at the respective primary antibody peaks whose Mean OD was higher than the assay LOD.

Variable	n	Min	Max	Median	IQR	Mean
S-IgG	106	0.321	1.947	1.074	0.742, 1.406	1.088
S-IgM	62	0.401	1.919	1.031	0.606, 1.329	1.000
S-IgA	51	0.226	1.518	0.486	0.339, 0.794	0.597
N-IgG	104	0.247	2.405	0.789	0.558, 1.267	0.951
N-IgM	52	0.090	0.726	0.182	0.130, 0.273	0.241
N-IgA	37	0.220	2.014	0.422	0.258, 1.401	0.797
RBD-IgG	85	0.140	1.326	0.555	0.355, 0.784	0.608
RBD-IgM	55	0.242	1.211	0.442	0.358, 0.691	0.549
RBD-IgA	48	0.107	1.756	0.254	0.159, 0.330	0.343

These were used as positive specimens for the computation of IgG, IgM, and IgA cut-off values for binding antibody detection.

**Table 2:** Displays the summary statistics of pre-COVID-19 negative specimens used to calculate optimal cut-off values for spike and nucleoprotein-directed IgG, IgM, and IgA binding antibodies.

Variable	n	Min	Max	Median	IQR	Mean
spike Protein: IgG Mean OD	205	0.000	1.146	0.081	0.045, 0.184	0.147
spike Protein: IgM Mean OD	206	0.000	1.406	0.158	0.077, 0.306	0.240
spike Protein: IgA Mean OD	206	0.000	0.737	0.00	0.00, 0.003	0.021
nucleoprotein: IgG Mean OD	207	0.000	1.843	0.236	0.158, 0.386	0.326
nucleoprotein: IgM Mean OD	207	0.000	1.107	0.256	0.154, 0.377	0.298
nucleoprotein: IgA Mean OD	207	0.000	1.093	0.093	0.056, 0.154	0.118
RBD-IgG	203	0.000	1.065	0.031	0.017, 0.067	0.060
RBD-IgM	203	0.000	1.374	0.142	0.086, 0.253	0.224
RBD-IgA	203	0.000	0.162	0.013	0.006, 0.024	0.017

### Determining antibody LOD and “limits of quantitation” using direct spike, RBD and nucleoprotein capture antigens

Assay limits (LOD and LOQ) were established in order to determine the smallest amount of analyte that can be reliably distinguished from analytical noise. Capture antigens (Spike, RBD, and nucleoprotein) were utilised to establish assay limits and specimen optical densities. Briefly, five ELISA plates each, coated with the spike, RBD, or nucleoprotein antigens were treated with serial dilutions of preestablished seropositive specimens with high OD values of matching antibody isotype (S-IgG, S-IgA, N-IgG, N-IgM, and N-IgA). Specimens were subjected to seven two-fold serial dilutions starting at 1:100 to construct 12 four-parameter logistic (4PL) standard curves per plate and 60 standard curves for each antigen-antibody combination. Utilizing the 420 net OD values and associated antibody concentrations from serial dilutions, a linear regression model was developed. Assay LOD values were computed using the method 3.3*(∂∂/S) while LOQ was computed using 10*(∂∂/S), where ∂∂ is the standard deviation of the intercept and S is the slope estimate and summarized in **Table 3**.

**Table 3:** ELISA “limits of detection” (LOD) and “limits of quantitation” (LOQ) using direct and indirect capture.

Direct detection Variable Antigen-specific	LOD	LOQ
S-IgG	0.310	0.940
S-IgM	0.383	1.160
S-IgA	0.217	0.658
N-IgG	0.213	0.647
N-IgM	0.089	0.271
N-IgA	0.215	0.651
RBD-IgG	0.121	0.366
RBD-IgM	0.238	0.720
RBD-IgA	0.106	0.321
Indirect detection Variable Kappa and Lambda	LOD	LOQ
spike: IgG	0.104	0.315
spike: IgM	0.168	0.509
spike: IgA	0.196	0.594
nucleoprotein: IgG Mean OD	0.105	0.317
nucleoprotein: IgM Mean OD	0.092	0.279
nucleoprotein: IgA Mean OD	0.182	0.550
RBD: IgG Mean OD	0.202	0.612
RBD: IgM Mean OD	0.344	1.043
RBD: IgA Mean OD	0.302	0.914

### Determining LOD and LOQ of antibody concentrations (ng/ml) using kappa and lambda indirect capture antibodies

Because antibody concentrations were estimated with an indirect method that uses commercial anti-human kappa and lambda standards as capture antibodies, assay limits for this procedure also used the indirect capture standards. Briefly, anti-human kappa and lambda capture antibodies were coated on five plates to determine the upper and lower limits of the standard curve, yielding a total of 60 standard curves. To generate 12 four-parameter logistic (4PL) standard curves per plate and 60 standard curves for each antigen-antibody combination, seven ten-fold serial dilutions of commercial IgG and IgA standards and seven five-fold serial dilutions of commercial IgM standards were performed, beginning at 1000 ng/ml. The 420 derived observations fitted on a linear regression model of net ODs and concentrations of the serially diluted standards. LOD and LOQ were determined using the formulas 3.3*(∂∂/S); where ∂∂ was the intercept standard deviation and S was the slope estimate, respectively, and summarized in **Table 3**.

### WHO Reference panels for assay verification

To ensure international comparability, the first international WHO reference standard for anti-SARS-CoV-2 antibody (NIBSC Code: 20/136) was used. This allowed the binding antibody values to be represented as arbitrary binding antibody unit (BAU). In order to compare tests detecting the same class of immunoglobulins with comparable specificity and antigenicity, the WHO standard was reconstituted to 1000 binding antibody units per millilitre, as previously described (22, 23). To calibrate the antibody concentrations from ng/mL to BAU/mL, WHO standards, commercial (IgG, IgM, and IgA) standards, and specimens were used. The WHO standard was diluted seven times (1:100 to 1:6400) to yield concentrations of 10, 5, 2.5, 1.25, 0.625, 0.313, and 0.156 BAU/mL. Commercial standards were diluted seven times (1:100 to 1:6400) to yield concentrations of 1000, 500, 250, 125, 62.5, 31.25, and 15.63 ng/mL. The tests were run on duplicate plates for three days in a row, yielding six replicates per test (24). To assess the assay’s ability to reliably distinguish positive and negative specimens, a WHO anti-SARS-CoV-2 IgG serology verification panel (NIBSC code: 20/B770) of 23 known positives and 14 known negatives was used

### Calibration of internal controls to WHO standards BAU/ml

To convert the concentrations in ng/mL into corresponding WHO standard, BAU/mL, a linear model was fitted. Statistical validity of the fitted model was evaluated by examining the parallelism and linearity of the WHO standard and the secondary standard over three days of assays using the coefficient of determination (R^2^). When the WHO standard, the secondary standard and the specimen models were linear and parallel, a final conversion factor was computed by averaging the conversion factors from days 1, 2, and 3.

### Establishing the assay dilution linearity

Linearity was determined for the Concentrations in the linear range of the calibration standard for S-IgG, S-IgM, S-IgA, N-IgG, N-IgM, and N-IgA respectively. Assay linearity was established by evaluating the linear range of concentrations of the calibration curve used to extrapolate antibody concentrations. A linear model was fitted to evaluate observed concentrations and expected concentrations for all the antibodies.

### Estimating IgG, IgM, and IgA binding antibody concentrations

Purified human IgG (Sigma, #12511) and IgA commercial standards (Sigma, #12636) at 10 and 5 mg/ml were reconstituted to 4.52 and 2 mg/ml, respectively, subjected to seven 10-fold serial dilutions ranging from 1000 to 0.001 ng/ml, and incubated alongside the test specimens. Purified human IgM (Sigma, # 18260) was reconstituted from 10 to 1 mg/ml and subjected to seven 5-fold serial dilutions ranging from 1000 to 0.06 ng/ml. The standards were incubated in duplicate wells pre-coated with 50µl of anti-human kappa and lambda light chain capture antibodies (Southern Biotech, #2060-01, #2070-01, 1:1 ratio, diluted 1:500). Optical density values from the standards were used to obtain a non-linear 4-parameter logistic (4-PL) modelled standard curve using the BioTek GEN5 software. Antibody concentrations were then extrapolated from the best linear range fit of the respective standard curves and corrected for the corresponding dilution factor. Concentrations below detection limit were assigned a of 0 ng/ml value.

### Cut-off values for spike and nucleoprotein IgG, IgM, and IgA OD positivity

**Figure 1:**
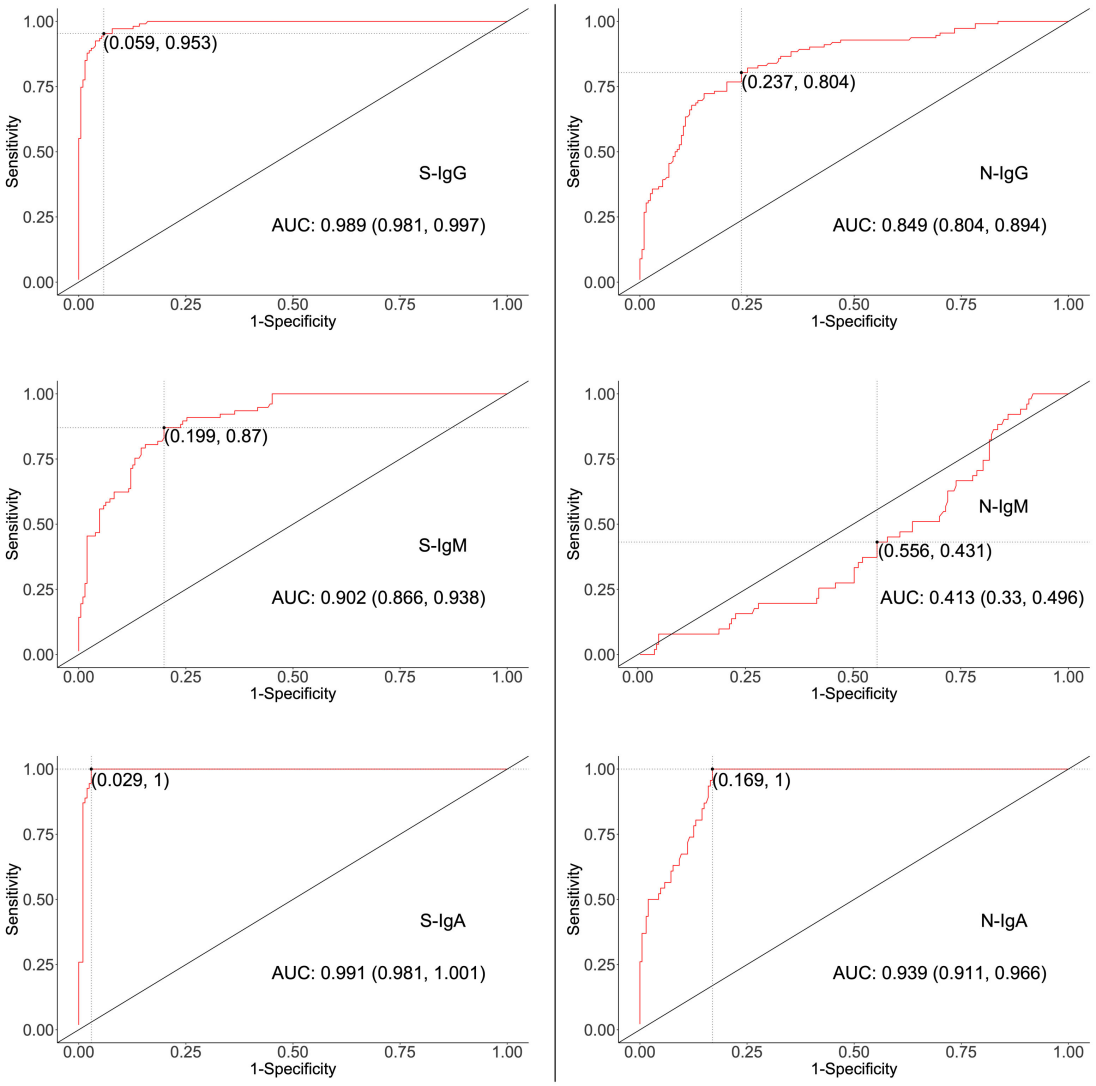
ROC curves for determining Cut-offs for spike- and nucleoprotein-directed antibodies. The two-dimensional algorithm ROC graph visualizes how well the test classifies subjects as positives or negatives for S- or N-directed IgG, IgM, or IgA antibodies. AUC; Area under the Curve, with 95% Confidence interval.

Five methods were used to determine cut-off values that best distinguished the presence of virus-specific IgG, IgM, and IgA in test specimens while prioritising specificity. Analyses included mean plus two standard deviations (mean ± 2SD), mean plus three standard deviations (mean ± 3SD), bootstrapping, receiver operator characteristic (ROC) analysis (**Figure 1**), and four-fold above blanks.

### Validating the in-house ELISA parameters

ELISA parameters were validated to assess detection reliability, including specificity, linearity, accuracy, inter-assay variation, precision between operators, and parallelism. Quantitation limits, including the “limits of detection” (LOD), “limits of quantitation” (LOQ), and limit of blank (LOB), were established to establish the minimum analyte concentration that can be reliably measured, as described by Shrivastava et al. (25) and Armbruster et al. (26). Parallelism was determined to assess relationships between optical densities derived from serum and plasma. WHO verification antibody panels 20/B770-02 and 20/136, monoclonal antibodies CR3022 and CR3009, and local plasmas with known reactivity (7, 9) were used in Levy-Jennings curves to monitor detection consistency over time. Inter-operator, intra- and inter-assay precision were monitored to ensure that OD450 values from repetitive tests conformed within the expected ≤ 25% coefficient of variation (CV). Further validation was done through an inter-site comparison of assays performed using identical specimens.

### Statistical analysis

Descriptive analysis was used to generate proportions for categorical data, while summary statistics were used for continuous variables. Correlations between continuous variables were computed using the Spearman rank correlations test; p-values ≤ 0.05 were considered statistically significant. All the Statistical analyses and graphical presentations were done with R Version 4.1, STATA 15, and GraphPad Prism version 9.40.

## Results

### Assay acceptance criteria

We developed an ELISA to detect SARS-CoV-2-specific IgG, IgM, and IgA optical densities at 450 nm as captured by spike, RBD, and nucleoprotein antigens. The assay simultaneously quantified IgG, IgM, and IgA antibody concentrations in ng/ml, captured by relevant specificities of anti-human kappa and lambda capture antibodies. The acceptance criteria for the test were CV between duplicate test wells of ≤ 25%, and an assay goodness of fit of R2 ≥ 0.9. Using linear regression to calibrate the derived concentrations to WHO standard BAU/mL units, the assay cut-off OD levels for anti-SARS-CoV-2 spike IgG, IgM, and IgA were equivalent to median antibody concentrations of 1.490723 (0.080429 - 5.099801), 3.164135 (1.477204 - 8.032694), and 0.00 (0.00 – 0.00) BAU/mL; nucleoprotein IgG, IgM, and IgA were equivalent to antibody concentrations of 3.271224 (2.301861 - 5.391706), 479.827 (265.4658 - 998.1329), and 136.7198 (39.48853 - 264.8783) BAU/ml; and RBD IgG, IgM, and IgA were equivalent to antibody concentrations of 2.403631 (0.834818 - 6.581954), 13.03752 (7.876224 - 24.78523), and 0.0534 (0.0534 - 35.31653)BAU/ml.

### Cut-offs for spike, RBD and nucleoprotein-directed IgG, IgM, and IgA assays

First, we explored mean ± 2SD and mean ± 3SD as the most straightforward approaches to computing cut-off values. Pre-pandemic specimens presumed to be of negative serostatus were used, and the upper limit was considered as the cut-off value. Thus, if a specimen’s optical density was above the upper limit, it was considered positive (**Table 4**). Next, the mean OD values of blanks from the various plates were used to calculate the 4-fold above blank cut-off point as 4*Mean (MnODblanks); any mean OD above the calculated cut-off point was classified as positive. Negative subjects were used to calculate the 95% confidence intervals using bootstrapping; the upper limit of the bootstrapped confidence interval (95% CI) was then chosen as the optimal cut-off value. Finally, we used the two-dimensional receiver operating characteristic (ROC) analysis of curves generated by plotting sensitivity (actual positive rate) against 1-specificity (false positive rate) for all possible thresholds to assess the performance of cut-off classifiers (**Figure 1**). Since ROC curve analysis requires both negative and positive specimens, PCR+ longitudinal specimens at the primary antibody peaks and pre-pandemic specimens above LOD were used. Optimal cut-off values were determined to maximise sensitivity while prioritising specificity by obtaining values whose 1- specificity and sensitivity were the closest to the point (0,1) on the ROC curve (minimum ER*(c)* function criteria) and assessing how proximal the area under the curve (AUC) was to a value of 1 (27, 28). Cut-off values based on the four-fold above blank, ROC, and bootstrapping approaches were computed and summarised in **Table 5**.

**Table 4:** Computation of Cut-off values using Mean +-2Sd and Mean +-3Sd.

Analysis	Antibody	# negatives	95% CI	Cut Off Point (Se, Sp)
Mean +-2 Standard Deviations	S-IgG	205	(-0.192, 0.487)	0.487 (92.45%, 95.12%)
	S-IgM	206	(-0.260, 0.739)	0.739 (69.35%, 95.14%)
	S-IgA	206	(-0.142, 0.185)	0.185 (100%, 97.09%)
	N-IgG	207	(-0.223, 0.874)	0.874 (45.19%, 93.24%)
	N-IgM	207	(-0.105, 0.701)	0.701 (3.85%, 95.17%)
	N-IgA	207	(-0.109, 0.345)	0.345 (62.16%, 98.07%)
	RBD-IgG	203	(-0.143, 0.264)	0.264 (87.06%, 96.55%)
	RBD-IgM	203	(-0.233, 0.681)	0.681 (27.27%, 93.60%)
	RBD-IgA	203	(-0.022, 0.057)	0.057 (100%, 97.54%)
Analysis		Variable	Mean ± 3SD	Cut-off Point (Se,Sp)
Mean ± 3 Standard Deviations	S-IgG	205	(-0.361, 0.656)	0.656 (82.08%, 98.05%)
	S-IgM	206	(-0.509, 0.989)	0.989 (51.61%, 98.06%)
	S-IgA	206	(-0.224, 0.267)	0.267 (92.16%, 99.03%)
	N-IgG	207	(-0.498, 1.149)	1.149 (30.77%, 98.55%)
	N-IgA	207	(-0.222, 0.458)	0.458 (48.65%, 98.55%)
	RBD-IgG	203	(-0.244, 0.365)	0.365 (72.94%, 98.03%)
	RBD-IgM	203	(-0.461, 0.910)	0.910 (12.72%, 97.04%)
	RBD-IgA	203	(-0.042, 0.077)	0.077 (100%, 98.03%)

Se, Sensitivity; Sp, Specificity.

**Table 5:** Calculated Cut Off Points using Boot strapping, ROC and 4-Fold Above Blanks.

Analysis	Antibody	# Negatives	Lower limit, Upper limit	Cut-off Point (Se,Sp)
Boot Strapping	S-IgG	205	(0.129, 0.167)	0.167 (100%, 73.17%)
	S-IgM	206	(0.213, 0.269)	0.269 (100%, 70.87%)
	S-IgA	206	(0.014, 0.031)	0.031 (100%, 83.98%)
	N-IgG	207	(0.297, 0.357)	0.357 (89.42%, 71.98%)
	N-IgM	207	(0.275, 0.323)	0.323 (19.23%, 67.63%)
	N-IgA	207	(0.105, 0.131)	0.131 (100%, 68.12%)
	RBD-IgG	203	(0.049, 0.073)	0.073 (100%, 76.85%)
	RBD-IgM	203	(0.199, 0.250)	0.250 (96.36%, 74.88%)
	RBD-IgA	203	(0.015, 0.020)	0.020 (100%, 70.44%)
Analysis		Variable	Mean ± 3SD	Cut-off Point (Se,Sp)
ROC	S-IgG			0.432 (96.23%, 94.15%)
	S-IgM			0.459 (93.55%, 86.89%)
	S-IgA			0.226 (100%, 97.57%)
	N-IgG			0.454 (82.69%, 79.71%)
	N-IgM			0.229 (42.31%, 44.44%)
	N-IgA			0.225 (97.30%, 87.44%)
	RBD-IgG			0.178 (96.47%, 94.58%)
	RBD-IgM			0.297 (90.91%, 78.82%)
	RBD-IgA			0.107 (100%, 99.01%)
Analysis		Variable	n (Blanks)	Cut-off Point (Se,Sp)
4-Fold Above Blanks	S-IgG	493		0.197 (100%, 77.07%)
	S-IgM	254		0.162 (100%, 51.94%)
	S-IgA	65		0.291 (86.27%, 99.03%)
	N-IgG	271		0.189 (100%, 32.37%)
	N-IgM	236		0.163 (61.54%, 27.54%)
	N-IgA	50		0.269 (70.27%, 93.72%)
	RBD-IgG	30		0.165 (96.47%, 94.09%)
	RBD-IgM	30		0.159 (100%, 57.14%)
	RBD-IgA	10		0.202 (66.67%, 100%)

### ROC analysis was the most optimal for computing cut-offs

**Figure 2:**
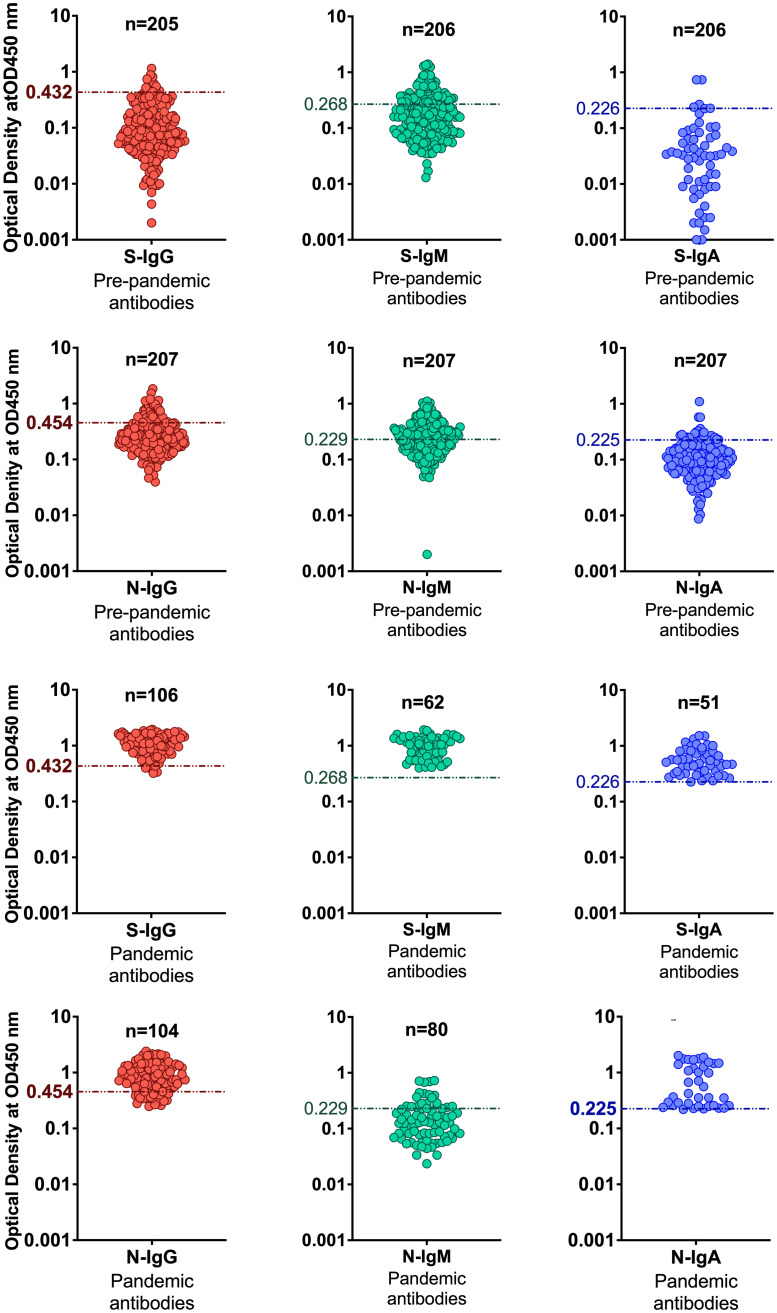
Distinguishing serostatus using ROC-derived cut-offs. The figure shows categorization of positive and negative specimens based on cut-off values determined by the ROC curve analysis method.

**Figure 3:**
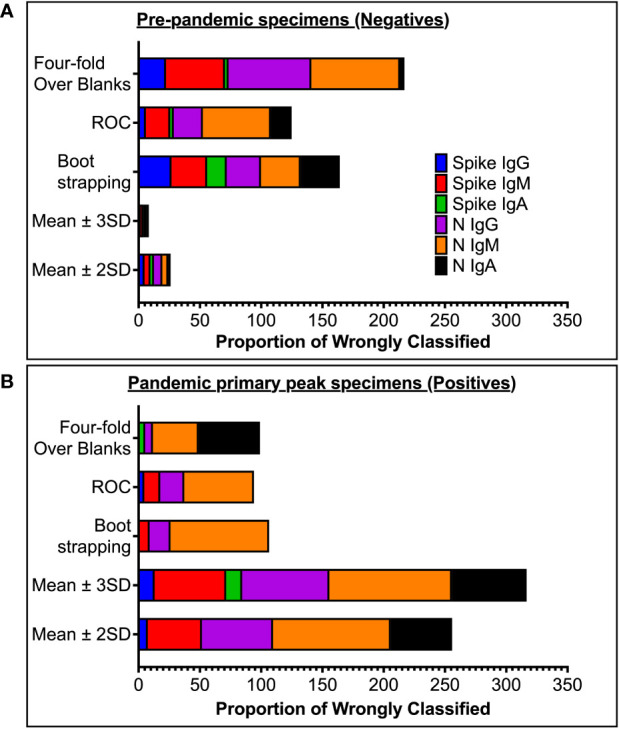
Proportion of wrongly classified specimen serostatus. **(A)** shows proportions of pre-pandemic (negative) specimens wrongly classified as positive (false positives). **(B)** shows proportions of antibody-peak specimens from PCR-confirmed subjects (positives) wrongly grouped as negative (false negative) using the five different methods of computing cut-off values.

High specificity was prioritized, and ROC analysis produced the best results in terms of sensitivity. As a result, the cut-off values for S- and N-directed IgG, IgM, and IgA antibody positivity, respectively, were adopted to be 0.432, 0.459, 0.226, and 0.454, 0.229, 0.225 (**Figure 2** and **Table 5**). Likewise, they were 0.178, 0.297, and 0.107 for RBD-directed IgG, IgM and IgA, respectively (**Table 5**). The sensitivity of mean ± 2SD and mean ± 3SD was much lower even though they had fewer false positive classifications than ROC (**Figure 3A**). ROC method had the lowest proportion of incorrectly classified specimens (false negatives), **Figure 3B**.

### Validating the in-house ELISA for accuracy and consistency

**Figure 4:**
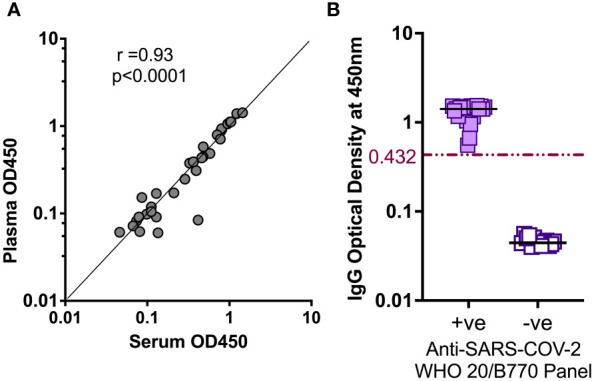
Validation of In-House ELISA parallelism and accuracy. **(A)** shows the parallelism of OD450 values from assays using serum and plasma of the same test subject. **(B)** shows the accuracy of the in-house ELISA verified against negative and positive specimens in WHO anti-SARS-CoV-2 verification standard panels. The ROC-derived cut-off of 0.432 accurately categorised the standard negative and positive specimens.

**Figure 5:**
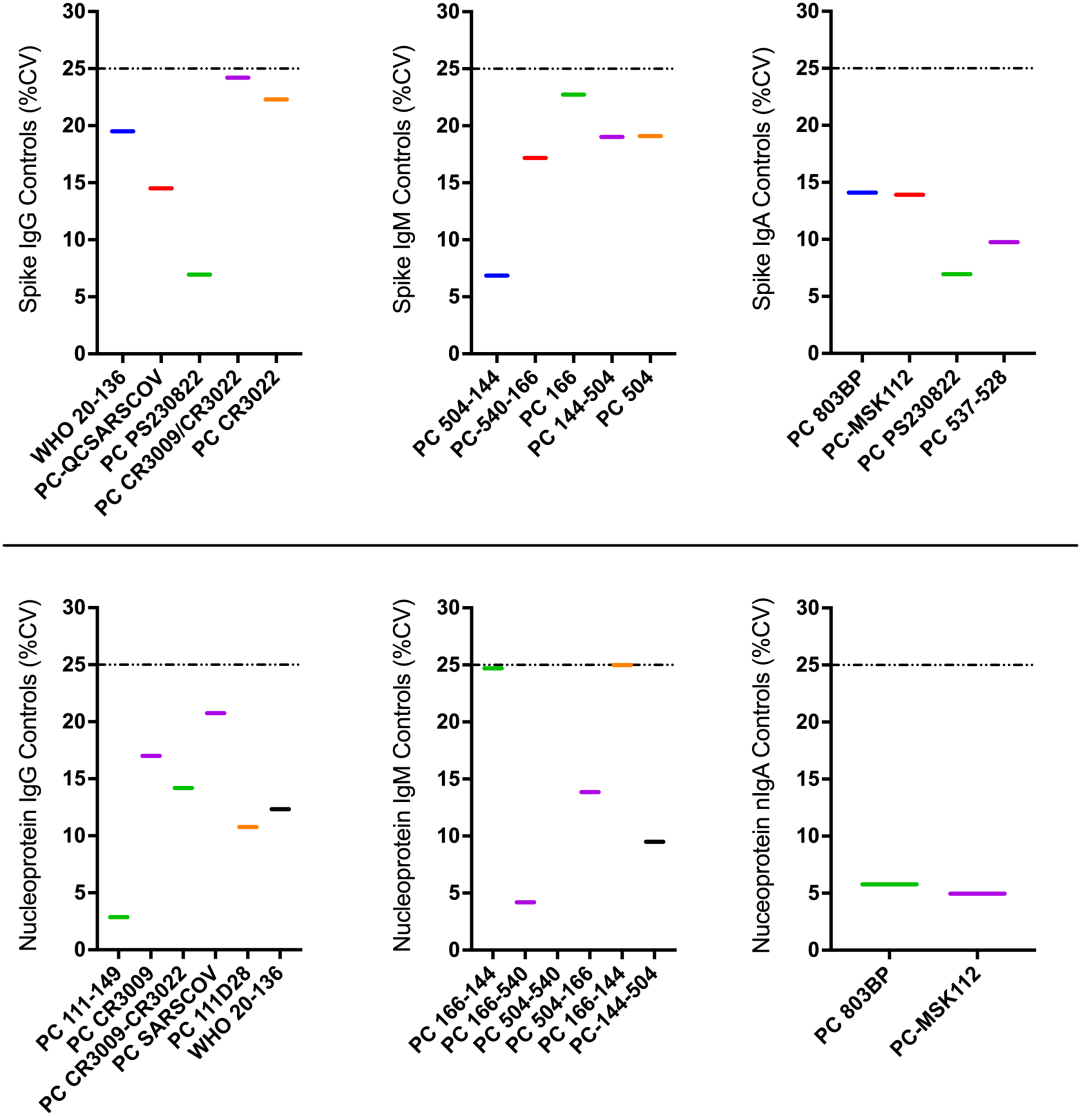
Illustrates inter-operator proficiency comparisons obtained by different operators testing identical control specimens repeatedly as part of the in-house ELISA. Colored horizontal bars represent the %CVs of concentrations for each quantified antibody-antigen specificity. The dotted horizontal lines represent the 25% CV proficiency cut-off.

**Figure 6:**
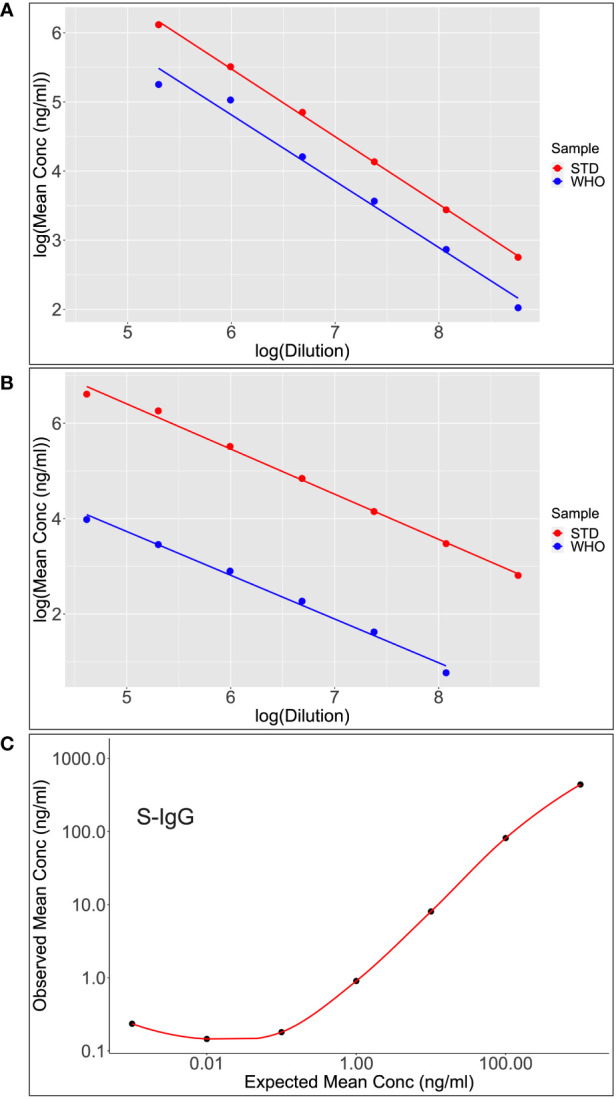
Shows scatter plots and lines of best fit for S-IgG **(A)** and S-IgA **(B)** following calibration of the WHO standard (WHO) against the assay commercial standard (STD). After analyzing the parallelism and linearity of the WHO and secondary standards linear models, a linear regression analysis was fitted to convert concentration in ng/ml to BAU/ml. Dilutional linearity was computed using a linear regression model to show the ability of the assay to provide results (Concentration(ng/ml) that are directly proportional to the expected Concentrations (ng/ml) at the various dilutions **(C)**.

**Figure 7:**
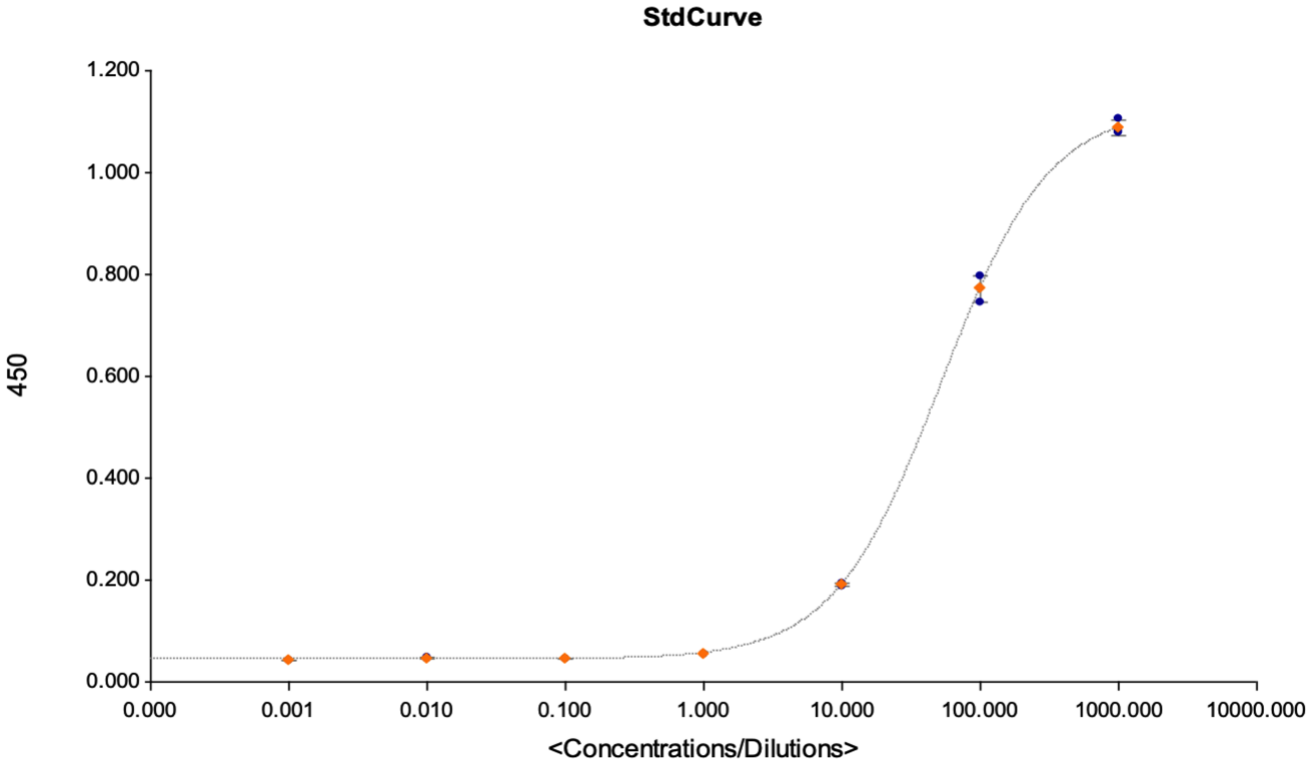
This figures shows the goodness of fit curves that were used as a criterion for accepting the assay for quantification of the detected antibodies. The graph is an illustration of a standard curve used for extrapolating antibody concentrations from OD450 values, and to calculate the assays LOD, LOQ and LOB at linearity R^2^ > 0.9.

**Figure 8:**
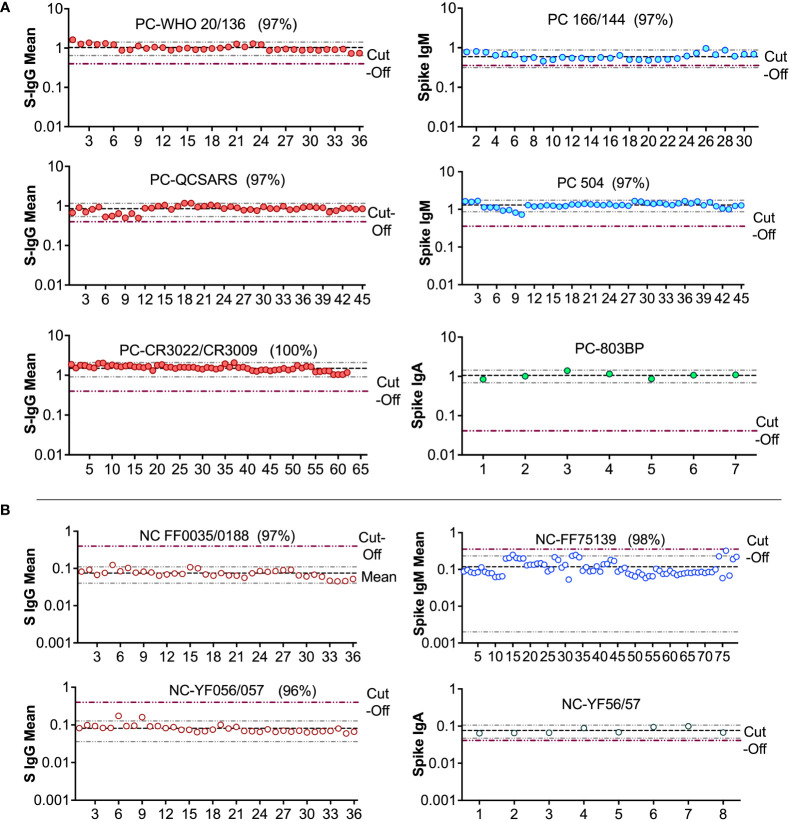
Levy Jennings charts plotted from a WHO international standard panel (20/136) and in-house local controls **(A)**: Levy-Jennings charts to track consistency of the in-house and international positive controls. **(B)** shows Levy-Jennings charts demonstrating the range of standard deviations from mean OD450 over time, with repeated use of local and WHO standard negative controls.

**Figure 9:**
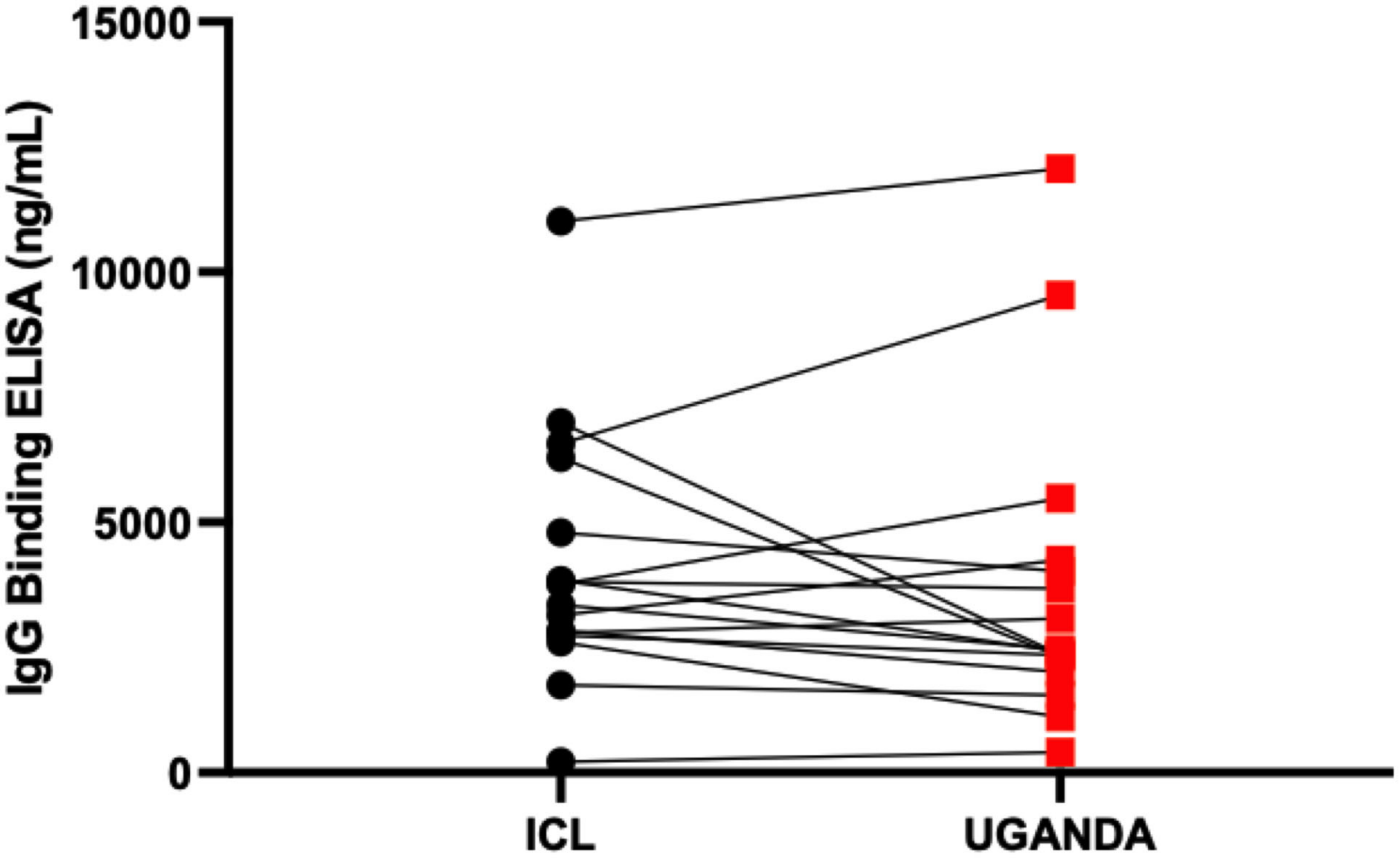
Inter-site cross-validation of the in-house ELISA. The figure compares antibody concentration concordance from use of the in-house ELISA to test the same specimens at Imperial College London and Uganda Virus Research Institute laboratories.

Parallelism was assessed by comparing plasma- and serum-derived test ODs. There was a strong positive correlation between ODs derived from testing plasma and serum (r = 0.93, p < 0.0001), Spearman’s correlation test (**Figure 4A**). Using international standards, S-IgG ODs were highly concordant regarding a WHO anti-SARS-CoV-2 verification panel comprising 23 positive and 14 negative specimens (lot 20/B770-02), yielding 100% specificity and sensitivity (**Figure 4B**). Inter-operator precision was obtained by different operators repeatedly testing identical specimens; the derived OD 450 nm values fitted within 25% CV across operators (**Figures 5**). Optical densities from repeated assays performed on different days were highly reproducible across tests and operators, yielding a median % CVs of 12.2 (IQR 8.7-16). After evaluating the parallelism and linearity of the WHO standard and the secondary standard, a linear regression analysis was performed. Concentration (in BAU/ml) = b0 + b1 * Concentration (in ng/ml) was the linear regression model that best fit the data (**Figures 6A, B**). Using the model in the equation above, the concentration in ng/ml was converted to BAU/ml based on the parameters b0 and b1 (**Table 6**). A linear model fitted to compare observed and expected concentrations yielded coefficients of determination (R^2^) >0.99 for all antibodies (**Table 6**). All antibodies achieved assay linearity, with a %CV ranging from 0.08% to 21.33% (**Figure 6C**). All standard curves used to extrapolate antibody concentrations met the assay’s goodness-of-fit acceptable criteria of R2 > 0.90 for all tests (**Figure 7**). Levy Jennings charts plotted from a WHO international standard panel (20/136) and in-house local controls consistently yielded ODs at CV 25% that were within two standard deviations of the mean, in conformation with the Westgard-Sigma rules (**Figures 8A, B**). Inter-site assay validation performed by testing the identical specimens at UVRI and Imperial College London laboratories resulted in a high antibody concentration concordance across sites, **Figure 9**.

**Table 6:** Table showing the Linear Regression Conversion Models to calibrate to WHO IS units and then assay linearity range for extrapolating concentrations.

Antibody	Linear Regression Model (BAU Conversion Model)	Linearity Range (ng/ml)	CV % Range	Adjusted R^2^
S-IgG	Conc _BAU/ml_ = 0.080429 + 0.018729 * Conc _ng/ml_	100 – 1.0	14.38% - 7.26%	1.00
S-IgM	Conc _BAU/ml_ = 0.465119 + 0.036897 * Conc _ng/ml_	1000 – 0.320	16.40% - 0.17%	0.9964
S-IgA	Conc _BAU/ml_ = -0.035959 + 0.190851 * Conc _ng/ml_	1000 – 8.0	18.82% - 0.08%	0.9944
N-IgG	Conc _BAU/ml_ = 0.252450 + 0.011651 * Conc _ng/ml_	100 – 1.0	21.33% - 0.33%	0.9999
N-IgM	Conc _BAU/ml_ = -1.808749 + 0.615273 * Conc _ng/ml_	1000 – 1.6	8.22% - 0.42%	0.9998
N-IgA	Conc _BAU/ml_ = 0.378230 + 0.622280 * Conc _ng/ml_	1000 – 8.0	17.39% - 0.09%	0.9953
RBD-IgG	Conc _BAU/ml_ = 0.834818 + 0.025888 * Conc _ng/ml_	1000 – 10.0	6.13% - 0.37%	0.9999
RBD-IgM	Conc _BAU/ml_ = 0.539426 + 0.031529 * Conc _ng/ml_	1000 – 1.6	12.32% - 0.04%	0.9980
RBD-IgA	Conc _BAU/ml_ = 0.053400 + 0.491130 * Conc _ng/ml_	1000 – 10.0	6.22% - 0.13%	0.9999

**Table 6** shows the linear Conversion models (column 2) for each of the antibodies. For S-IgG, the linear model is Conc (in BAU/ml) = 0.080429 + 0.018729 * Conc (in ng/ml). This implies given a spike IgG Concentration of 1000 ng/ml, the above linear model can be used to get the equivalent spike IgG Concentration in BAU/ml as 0.080429 + 0.018729 * 1000 = 18.80943 BAU/ml. Columns 3, 4 and 5 show that linearity rages were achieved for all antibodies, their associated %CV and coefficient of determination (R^2^) are indicated.

## Discussion

In this study, we optimised and validated an in-house SARS-CoV-2 spike, RBD and nucleoprotein IgG, IgM and IgA binding antibody ELISA that is relevant for serosurveillance studies in populations primarily comprised of asymptomatic and mildly symptomatic COVID-19 cases (29). Previously, similar efforts (30–32) to develop SARS-CoV-2 binding antibody ELISAs have been limited by the heavy reliance on hospitalised patients with severe disease as the positive controls in determining cut-off threshold values. Such criteria are sub-optimal in Sub-Saharan Africa and similarly affected regions, where SARS-CoV-2 infection has mostly been asymptomatic or accompanied by mild symptoms because the resulting threshold values would be so stringent as to preclude detection of mild infection, leading to a high false-negativity rate (33). Use of severely infected, highly symptomatic PCR-confirmed positive control subjects inadvertently skews the sensitivity of the assay, since those who are asymptomatic or mildly symptomatic usually have much lower and sometimes conventionally undetectable levels of antibodies (34, 35). Given the SARS-CoV-2 rt-PCR-confirmed positive nature of the specimens used in this study, which included 110 (66%) asymptomatic and 25 (15%) mildly symptomatic study participants, the defined criteria herein are thus optimized for use in Sub-Saharan Africa and similarly affected settings.

Furthermore, by utilizing an indigenous population to determine cut-off threshold values, the optimized ELISA accounted for the possibility of serological cross-reactivity to common coronaviruses and other unknown antigens, which is an important confounding factor in the development of SARS-CoV-2 serological tests (12). As a result, this optimised ELISA enables the distinction between infected and non-infected individuals more accurately, providing more reliable results while also improving our understanding of the epidemiology of SARS-CoV-2 infection. Cut-off values cannot be universal and must be determined to suit the baseline clinical profile of the population under consideration (27). In other parts of the world, where an asymptomatic and mild phenotype has not been the predominant clinical feature of COVID-19, a different cut-off value might be more suited. Thus, the cut-off values determined herein are relevant to Sub-Saharan Africa and other regions where there are perceived similar pre-existing cross-reactive antibody responses from previous encounters with other coronaviruses (12, 14), and populations where asymptomatic and mildly symptomatic cases have primarily characterised the COVID-19 pandemic.

Following comparative statistical analyses of five of the various approaches used in determining cut-off values for categorising positive and negative specimens, we found the ROC curve analysis approach to be the most optimal at maximising sensitivity and prioritising specificity of the assays. Whereas some similar studies (1, 15) used the more intuitive methods of mean ± 2SD or mean ± 3SD, we found these methods to have high proportions of the PCR-confirmed specimens wrongly classified as negative and remarkably lower overall sensitivity compared to other methods (36). Furthermore, the reliance on the mean would require normal data distribution. In our case, the non-normally distributed data further weighed against these and similar methods of determining cut-offs. Compared to the 4-fold above blanks and bootstrapping methods, the ROC curve analysis approach had a higher specificity, which was of high priority. Consequently, we chose to use more precise methods of calculating diagnostic local cut-offs based on data from the receiver operating characteristic (ROC) curve. Therefore, our data supported ROC as the optimal method for the detection of IgG, IgM, and IgA antibody cut-off threshold values, at specificities of 94, 80, 97 (spike), 95, 84, 100 (RBD); and 95, 84, 100 (nucleoprotein), respectively. WHO classifies anti-SARS-CoV-2-S1-RBD IgG levels of 44–53 BAU/mL, 200–300 BAU/mL, and 700–800 BAU/mL as low, mid, and high titers, respectively (22). Thus, our S-IgG negative specimens’ concentration at cutoff value of 1007.0 ng/ml (18.94 BAU/mL), and our RBD-IgG negative specimens’ equivalent median concentration of 2.40 (0.835 - 6.58 BAU/ml) anti-SARS-CoV-2-S1-RBD IgG is consistent with WHO’s established low titer estimates.

Despite comparing five methods for determining N-IgM, the main limitation of this study was that the detection threshold was low across all tests, with low sensitivity and specificity (AUC < 0.5). The low levels of N-IgM antibody detected in all PCR-confirmed specimens significantly confounded their differentiation from negative specimens. Our results suggest that the N-IgM assay alone is insufficient for the laboratory diagnosis of SARS-CoV-2 infections, particularly in populations with low antibody levels during the early stages of infection. However, this test shows promise as a faster and less expensive alternative to RT-PCR for the initial screening of individuals who might be infected with COVID-19. In clinical practice, it is recommended to combine N-IgM with other laboratory tests, such as viral antigen and RNA detection, to improve the accuracy of SARS-CoV-2 infection detection and optimize the early detection of SARS-CoV-2 infections. Further optimization of N-IgM thresholds in various groups and stages of COVID-19 infection is required for improved diagnostic utility.

The WHO standards were clearly defined as spike IgG-positive positive control specimens. Unfortunately, there are no positive controls for other antigens and antibody isotypes. In the absence of positive controls, we needed a starting point and thus selected all samples at the peak of each antibody isotype, some of which were marginally positive. This is more evident in the IgM and IgA assays using the N protein as the capture reagent. There is a need for more precisely defined antigen and antibody isotype controls to guide the establishment of assay limits.

Lastly, the primary antibody-peak specimens used in the assay validation were primarily collected during the A23.1 variant wave in Uganda, and the S, N, and RBD proteins used correspond to the Wuhan variant of the wild type. A phylogenetic analysis of the A23.1 genomic sequences in Uganda identified four amino acid substitutions in the spike protein (37). Despite some similarities with other variants, the mutations are fewer than those found in other variants of concern, such as Omicron. While the assay limits reported apply to less mutated virus specimens, the evolution and emergence of new variants is a potential confounding factor for serological profiling. The authors acknowledge that SARS-CoV2 has mutated into several variants and sub-variants, including Alpha, Beta, Delta, and Omicron, with the latter being more prevalent in the population. Omicron sub-variants are highly mutated versions of the Wuhan, which may result in a distinctive serological profile compared to the Wuhan. While the assay limits remain relevant for several ongoing clinical trials based on the Wuhan prototype, they will need to be re-evaluated considering the epidemic’s mutated strains.

In conclusion, in the absence of a gold standard for SARS-CoV-2 antibody immunoassays (10), especially for Sub-Saharan Africa and similarly affected regions, we present a highly accurate and consistent in-house ELISA, having been statistically validated in at least two sites and verified against a panel of WHO international standard specimens. In the assays, using either serum or plasma would give similar results as found in our parallelism analysis. Furthermore, the assay can be adapted for use in the settings of Sub-Saharan Africa for immunosurveillance, profiling the course of antibody responses to SARS-CoV-2 and COVID-19 vaccines, accurate tracking of the pandemic burden, and assessing the risk of future infection/reinfection in a given population. However, the low specificity and sensitivity of N-IgM suggest that the gold standard of RT-PCR testing is still necessary for accurate diagnosis.

## Data availability statement

The raw data supporting the conclusions of this article will be made available by the authors, without undue reservation.

## Ethics statement

The studies involving human participants were reviewed and approved by the Uganda Virus Research Institute (UVRI) Research and Ethics Committee (Ref: GC/127/833) and the Uganda National Council of Science and Technology (Ref: HS637ES). The patients/participants provided their written informed consent to participate in this study.

## The COVID-19 Immunoprofiling Team

Claire Baine, Jackson Sembera, Susan Mugaba, Christine Hermilia Akoli, Geoffrey Odoch, Deus Mwesigwa, Peter Ejou, Joseph Ssebwana Katende.

## Author contributions

Conceptualization, design, analysis, interpretation of the work: JS; Design, data acquisition and analysis, drafting and revision of the manuscript: PN, GKO; Data acquisition PN, BG, LK, AN, The COVID-19 Immunoprofiling team; Preliminary analyses VA, JS, PK, GKO; Statistical analysis and interpretation of data: VA, JS, PK. Original Draft: GKO, PN, JS. Reviewing and Editing: PK, JF, MC. Funding Acquisition: MM, JS, JF, MC. Scientific oversight, and approval of the content: JS, PK. All authors read and approved the final manuscript.
